# Student Experience with a Quality Improvement Project in the Emergency Department

**DOI:** 10.5811/westjem.2018.8.39183

**Published:** 2018-09-10

**Authors:** Charlotte Burford, Alexandra von Guionneau

**Affiliations:** King’s College London, Faculty Life Sciences and Medicine, London, United Kingdom

Dear Editor,

We read with great interest Manning et al.’s recent article on the use of medical student quality improvement projects (QIP) to promote evidence-based care in the accident and emergency (A&E) department (Manning et al., 2018). We believe that students are well positioned to effect change via QI initiatives and offer our experience to support their recommendations, alongside further suggestions to aid implementation and integration of medical student QIPs into clinical practice.

As part of our penultimate-year curriculum, we designed and managed a six-month QIP in a district general teaching hospital in southern England, whose trust receives approximately 138,000 visits per year (Western Sussex NHS Foundation Trust, 2017). While Manning et al. focused on implementing treatment pathways, our project focused on improving patient flow in a triage area on the acute medical ward, receiving referrals from community general practitioners, as well as less acutely unwell patients from the A&E department.

In their article, Manning et al. highlighted bidirectional alignment, “the idea that an institutional problem should be evaluated and addressed from the bottom-up as well as the top-down,” as an important part of the QI process. Our experiences echo this; spending time immersed in the clinical area observing frontline staff drastically altered our perception of the patient flow process and informed our root-cause analysis of the barriers to efficient patient flow. Our first recommendation for any medical student QIP would be to pair students with clinical champions for change management who are instrumental in the day-to-day delivery of services. This will ensure projects are tailored and address the real issues impacting staff and patients alike.

Despite identifying and recruiting champions for change management, we faced significant challenges in implementing improvements. As students, we lacked autonomy to enact change in clinical areas. Although this proved to be a useful learning experience in independently raising concerns and proposing solutions, our second recommendation would be to pair students with a senior clinical practitioner in the department who is able to lend authority to students to undertake changes in the clinical setting. Involving both a senior leader and frontline staff will not only support students but, more importantly, encourage sustainable change which will outlast the duration of the student project.

Although we recommend a close working relationship with staff, we believe as external third-party observers that we were able to offer a unique perspective in the QI process. Manning et al. describe the “fresh perspective” of medical students as influential in creating “novel solutions.” Indeed, we observed that frontline staff were aware of the main barriers to patient flow, yet appeared blinded to obvious solutions, remaining entrenched in current practice. For example, we identified a lack of clinical space as a barrier to flow and raised the possibility of using existing curtains to form temporary bays within the waiting room. This realization surprised staff, and one member of staff even suggested we should take them down completely to save space rather than utilizing them for clinical workspace. A fresh perspective is of particular importance in an acute medical setting where the clinical demands on staff hardly allow for lunch breaks, let alone detailed reflection and analysis of clinical practice. The two-day training we received in QIP methodology taught us how to use lean management tools to identify problem areas within the flow process: developing a root-cause analysis, plan-do-study-act cycles, A3 problem solving, engaging the key stakeholders, and establishing a plan for measuring the outcomes of our QI initiative. Furthermore, this training facilitated proactive inter-professional communication, more so than our conventional interprofessional education.

Our third recommendation would be for students to receive training, whether in the form of classroom-based teaching or online modules, before commencing clinical QIPs. This will ease their integration within the clinical team and reduce demands on staff, facilitating a more successful QIP.

Overall we strongly agree with the findings of Manning et al. and believe that QIPs have given us a unique insight into how we can effect evidence-based change in a clinical environment, which has real world implications on a day-to-day basis. We would encourage all A&Es to reinforce the role of medical student QIPs in improving patient care.
